# Neurexin in Embryonic *Drosophila* Neuromuscular Junctions

**DOI:** 10.1371/journal.pone.0011115

**Published:** 2010-06-14

**Authors:** Kaiyun Chen, Elena O. Gracheva, Szi-Chieh Yu, Qi Sheng, Janet Richmond, David E. Featherstone

**Affiliations:** Biological Sciences, University of Illinois at Chicago, Chicago, Illinois, United States of America; Medical College of Georgia, United States of America

## Abstract

**Background:**

Neurexin is a synaptic cell adhesion protein critical for synapse formation and function. Mutations in neurexin and neurexin-interacting proteins have been implicated in several neurological diseases. Previous studies have described *Drosophila* neurexin mutant phenotypes in third instar larvae and adults. However, the expression and function of *Drosophila* neurexin early in synapse development, when neurexin function is thought to be most important, has not been described.

**Methodology/Principal Findings:**

We use a variety of techniques, including immunohistochemistry, electron microscopy, in situ hybridization, and electrophysiology, to characterize neurexin expression and phenotypes in embryonic *Drosophila* neuromuscular junctions (NMJs). Our results surprisingly suggest that neurexin in embryos is present both pre and postsynaptically. Presynaptic neurexin promotes presynaptic active zone formation and neurotransmitter release, but along with postsynaptic neurexin, also suppresses formation of ectopic glutamate receptor clusters. Interestingly, we find that loss of neurexin only affects receptors containing the subunit GluRIIA.

**Conclusions/Significance:**

Our study extends previous results and provides important detail regarding the role of neurexin in *Drosophila* glutamate receptor abundance. The possibility that neurexin is present postsynaptically raises new hypotheses regarding neurexin function in synapses, and our results provide new insights into the role of neurexin in synapse development.

## Introduction

The synaptic clefts of both mammalian central glutamatergic synapses and *Drosophila* NMJs are approximately 20 nanometers wide and contain extracellular domains of cell adhesion proteins and associated molecules [1], [2], [3], [4]. A number of synapse-specific cell adhesion molecules, including neurexin, have been identified, and several have been proposed to be important for triggering synapse formation and serving as anchors for aggregation of other synaptic molecules [5], [6], [7].

Neurexin first attracted attention as a receptor for alpha latrotoxin, a black widow spider venom component that causes massive neurotransmitter release [8], [9]. Recent genetic studies have linked mutations in human neurexin to autism, mental retardation, and schizophrenia [10], [11], [12], [13], [14], [15], [16], [17], [18], [19]. Thus, neurexin clearly plays an important role in nervous system development and function.

The precise role of neurexin has been difficult to study due to the protein's complexity. Mammalian genomes contain three neurexin genes. Each gene drives expression of two primary neurexin isoforms via alternative promoters: a full-length (alpha) neurexin, and a short (beta) neurexin that contains a common C-terminus [8], [20], [21]. Each of these six primary mammalian neurexin transcripts can then be alternatively spliced to form potentially thousands of different proteins [22], [23].

Despite this complexity, important progress has been made. Neurexin has been shown to bind to several key synaptic proteins, including the synaptic vesicle protein synaptotagmin, the synaptic scaffolding proteins Mint and CASK, and the cell adhesion molecules neuroligin and LRRTM2 [24], [25], [26], [27], [28]. Neurexin is thought to nucleate aggregation and organization of synaptic components around a transynaptic cell adhesion complex. Consistent with this, neurexin-neuroligin and neurexin-LRRTM2 interactions promote synapse formation and/or aggregation of synaptic molecules [28], [29], [30], [31], [32], [33], [34], [35].

The first complete knockout of neurexin was achieved in *Drosophila*, which appears to possess only a single relatively simple neurexin similar to mammalian alpha neurexin [36], [37]. Surprisingly, *Drosophila* neurexin null mutants are viable and fertile (albeit with reduced lifespan), but show reduced synapse number and impaired synaptic transmission [36], [37]. Behavioral tests also reveal impairments in larval learning [36].

Here, we extend previous studies by introducing novel *Drosophila* neurexin mutant alleles and examining neurexin mutant synapses in embryonic *Drosophila* during the NMJ formation and the initial wave of synaptogenesis. Synapse loss is an important part of normal nervous system development [38], and the reduced synapse number previously observed in neurexin mutants could represent impairment in initial synapse formation or destabilization and subsequent loss of synapses. Our study addresses this question directly. We also consider whether *Drosophila* neurexin might function postsynaptically as well as presynaptically, and provide more detail regarding the role that neurexin plays with regard to postsynaptic glutamate receptor abundance in *Drosophila* neuromuscular junctions (NMJs).

## Results

**Figure 1:**
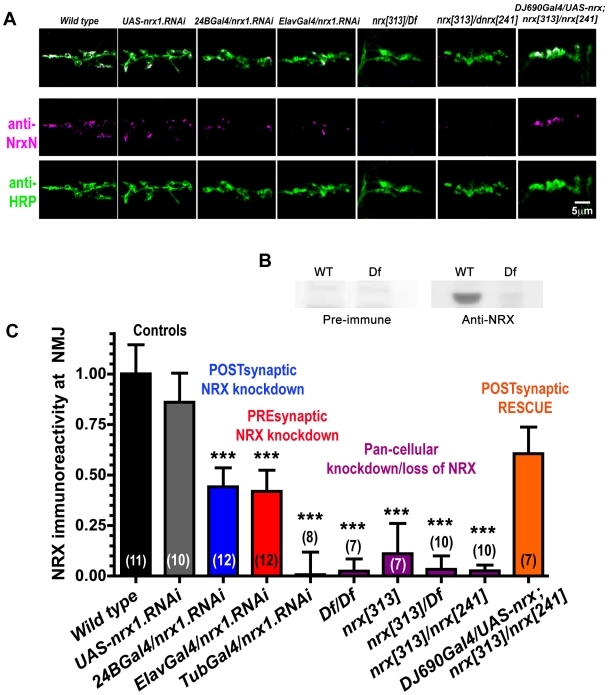
Neurexin expression in embryonic NMJs after specific genetic manipulations. A: representative confocal micrographs of embryonic muscle 6/7 NMJs stained with antibodies against the N-terminus of neurexin (magenta) and HRP (green). B: Neurexin immunoreactivity on western blots is absent in preimmune negative controls and in *Df(3R)Exel6191*, a genomic deletion that completely removes the neurexin locus. C: quantification of neurexin immunoreactivity from images like those shown in A. Wild type and *UAS-nrx1.RNAi* are controls. *24BGal4/nrx1.RNAi* and *ElavGal4/nrx.RNAi* represent expression of neurexin RNAi in muscles and neurons, respectively. *TubGal4/nrx1.RNAi* represents expression of neurexin RNAi in all tissues. Df represents *Df(3R)Exel6191*. *nrx[313]* and *nrx[241]* are neurexin mutant alleles. *DJ690Gal4/UAS-nrx;nrx[313]/nrx[241]* represents both muscle and neuron expression of neurexin cDNA in a *nrx[313]/nrx[241]* mutant background. Numbers in parentheses represent N, the number of animals examined.

We isolated several independent *Drosophila* neurexin mutant alleles by mobilization of *P{Mi}Nrx-1{MB00002}*, a minos P-element in the *Drosophila* neurexin gene. One of these alleles was *nrx[313]*, which we selected for further study because it appeared to be a null allele (see below). Previous studies [37], [39] have shown that *Drosophila* neurexin is highly abundant in mature larval neuromuscular junctions (110–120 h after egg laying, AEL). Using independently generated anti-neurexin antibodies (see methods), we confirmed the presence of neurexin in larval NMJs, and extended this analysis to embryonic NMJs (22–24 h AEL; Fig. 1). As expected, we found that neurexin immunoreactivity was also abundant in embryonic NMJs (Fig. 1). Neurexin NMJ immunoreactivity was eliminated in embryos homozygous for *Df(3R)Exel6191*, a deficiency that completely removes the neurexin gene (Fig. 1). Neurexin NMJ immunoreactivity was also eliminated in homozygous *nrx[313]* mutants, in *nrx[313]/Df(3R)Exel6191* mutants, and in *nrx[313]/nrx[241]* mutants (Fig. 1), where *nrx[241]* is a previously-described small deficiency that deletes the *neurexin* coding region [37]. Neurexin immunoreactivity in NMJs could be restored in *nrx[313]/nrx[241]* mutants by transgenic expression of a neurexin cDNA (*DJ690Gal4/UAS-nrx;nrx[313]/nrx[241]*). Taken together, these data suggest that our antibody is specific for neurexin in embryonic NMJs, that neurexin is abundant in embryonic NMJs, and that *nrx[313]* is a protein null allele or very strong hypomorph.

Transgenic expression of neurexin RNAi also reduced neurexin immunoreactivity in embryonic NMJs (Fig. 1). Strong ubiquitous expression of neurexin RNAi (*TubGal4/nrx.RNAi*) reduced neurexin immunoreactivity in embryonic NMJs to undetectable levels, comparable to *nrx[313]* or the neurexin gene deletions *Df(3R)Exel6191* and *nrx[241]* (Fig. 1). However, we could only eliminate half the neurexin NMJ immunoreactivity by strong neuronal (presynaptic) expression of neurexin RNAi (*ElavGal4/nrx1.RNAi*; Fig. 1). Surprisingly, we were also able to eliminate half the neurexin NMJ immunoreactivity by muscle-specific (postsynaptic) expression of neurexin RNAi (*24BGal4/nrx1.RNAi*; Fig. 1). The simplest conclusion, based on these data, is that neurexin in *Drosophila* embryonic NMJs is expressed both pre and postsynaptically.

**Figure 2:**
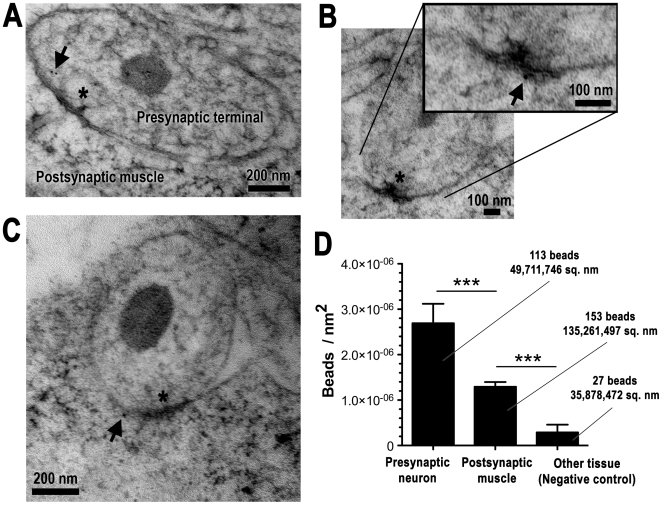
Immuno electron micrographs from embryos showing neurexin protein immunoreactivity in both presynaptic terminals (A), and in postsynaptic muscle (B,C). Synapses (marked by asterisks) are indicated by the presence of electron dense membrane and, in many cases (panels A, B, B inset), by distinctively-shaped presynaptic electron dense structures (‘T-bars’). Arrows in each panel point to 10 nm beads indicating positive neurexin immunoreactivity. D: Average density of 10 nm beads in neuronal axon terminals, body wall musculature, and other tissue (cuticle, gut, or bacteria surrounding the animal during HPF), calculated from each section. The total number of beads counted, and the total area of each tissue examined across all sections, is also indicated.

To further explore the idea that neurexin might be expressed in postsynaptic muscle cells, we analyzed neurexin immunoreactivity in embryonic NMJs using immuno electron microscopy following high-pressure freeze fixation and freeze substitution (HPF/FS) (Fig. 2). In one set of experiments, we counted 293 beads in 24 NMJ sections from 2 different embryos. Most immunoreactivity was found in presynaptic terminals (Fig. 2A, D). However, a considerable amount of immunoreactivity was also found in postsynaptic muscle (Fig. 2B–D). We measured nonspecific background immunoreactivity in the same sections by counting beads in all other ‘negative control’ tissues (gut, cuticle, fat bodies, or bacteria surrounding animals during HPF). As expected, very little immunoreactivity was observed in negative control tissue. To ensure that the beads we observed in muscle did not represent spurious background, we analyzed another 100 sections from 12 different NMJs in 3 more animals under higher stringency/lower antibody conditions. Under these conditions, no beads were observed in negative control tissues, but the number of beads in muscle compared to nerve terminal was proportionally the same as measured under the low stringency conditions. Specifically, 14 of 21 beads (67%) were in presynaptic terminals, and 7 of 21 beads (33%) were in postsynaptic muscle. These data, like the immunohistochemical data after RNAi, suggest that neurexin protein near embryonic NMJs is present in both presynaptic nerve terminals and postsynaptic muscle.

**Figure 3:**
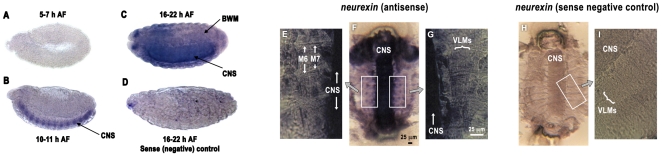
Neurexin mRNA expression in *Drosophila* embryos, detected using RNA in situ hybridization. A: *Neurexin* mRNA expression in *Drosophila* embryos. RNA in situ hybridization reveals no detectable *neurexin* mRNA expression early in embryogenesis (5-7 h after fertilization/AF). B: As neuronal differentiation proceeds at 10-11 h AF, however, *neurexin* mRNA expression is detectable throughout the central nervous system (CNS). C: Late in embryogenesis (16-22 h AF), during early neuromuscular junction formation, *neurexin* mRNA is abundant within both the CNS and faintly visible in body wall muscles (BWM). E-G: Shown are filleted whole embryos (F, H) and higher magnification images of the body wall musculature (E, G, I). Staining (dark blue color, representing neurexin expression) was abundant in the central nervous system (CNS) and body wall muscles. The positions of ventral longitudinal muscles (VLMs) and muscles 6 and 7 (M6, M7) are indicated by white arrows. All NMJ experiments described in this paper were performed on muscle 6/7 NMJs. Note the lack of staining in CNS and BWM in the sense negative controls (H,I).

If *Drosophila* neurexin is expressed in postsynaptic body wall muscles, then we should be able to detect neurexin expression in muscles using RNA in situ hybridization. We therefore performed RNA in situ experiments on whole embryos (Fig. 3). Consistent with previous reports [36], [37], we saw strong neurexin expression in the nervous system (Fig. 3). However, in late-stage embryos after body wall muscle formation we also saw weak body wall muscle expression of neurexin (Fig. 3C). Because cuticle formation in late-stage embryos can limit detection of muscle gene expression, we also examined neurexin RNA expression in filleted late-stage embryos (Fig. 3E–F). Neurexin expression was prominent in the nervous system of filleted embryos, consistent with strong nervous system expression of neurexin and the fact that the nervous system is many cell layers thick. However, neurexin expression was also detectable in body wall muscles (Fig. 3E), including individual ventral longitudinal muscles 6 and 7 – the same muscles that form the postsynaptic side of the NMJs examined immunohistochemically (Fig. 1).

**Figure 4:**
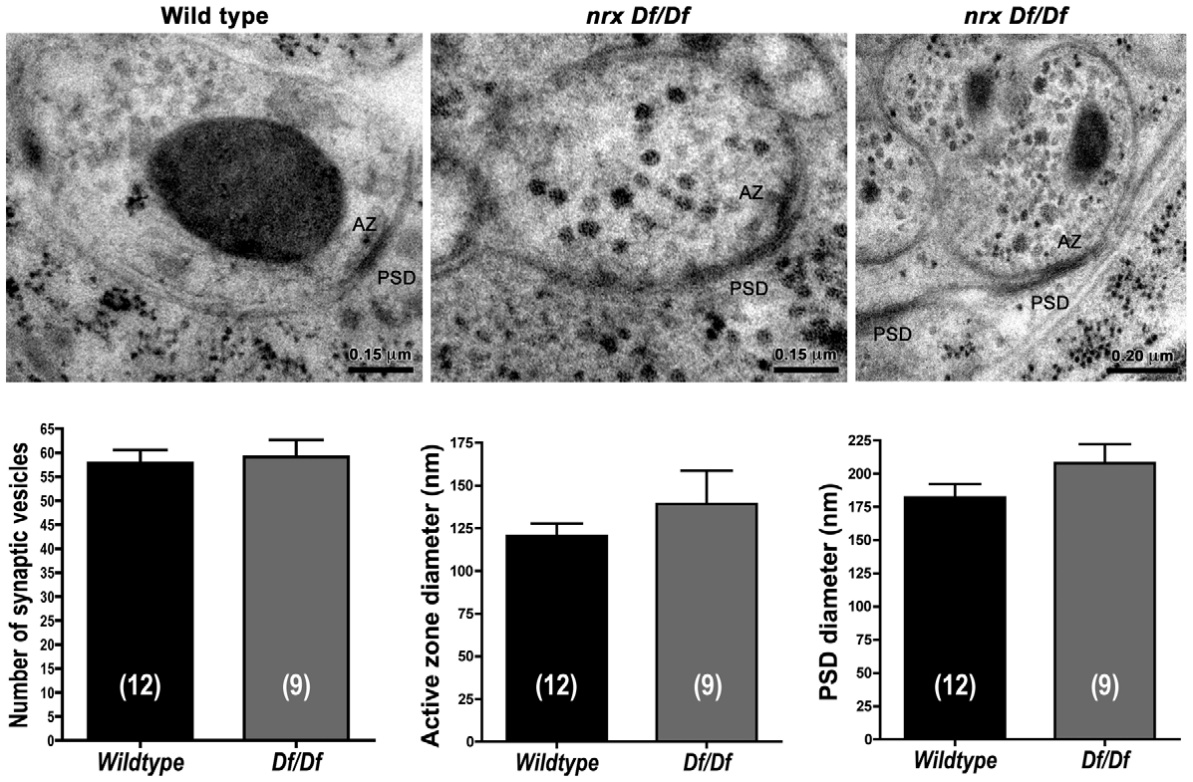
Embryonic synapse ultrastructure in the absence of neurexin. Homozygous *Df(3R)Exel6191* mutants (abbreviated ‘*Df/Df’*) are devoid of neurexin. Synaptic vesicles, electron dense presynaptic membrane and presynaptic dense projections at active zones (AZ) are visible in both wildtype and homozygous *Df(3R)Exel6191* mutant embryonic body wall NMJs. Similarly, both WT and *Df(3R)Exel6191* mutant NMJ synapses show postsynaptic electron dense membrane (PSD). NMJ synapses in *Df(3R)Exel6191* mutants show no significant changes in the number of synaptic vesicles, diameter of AZs, or diameter of PSDs, compared to wildtype NMJ synapses. However, the electron dense postsynaptic muscle membrane in *Df(3R)Exel6191* mutants sometimes extends beyond the presynaptic specialization, or is not associated with any visible presynaptic specialization. In some cases, we serial sectioned individual synapses to confirm that apparently misaligned PSDs did not pair with an AZ in a neighboring section. 142 sections from several animals were analyzed for the active zone & PSD statistics. For quantification, measurements were made from all available sections of a synapse and then averaged together to produce a single measurement for each synapse. N (indicated in figure) represents the number of unique NMJs that were sectioned.

We next examined the form and function of embryonic NMJs after loss of neurexin. Previous reports [36], [37] showed that neurexin is not essential for NMJ formation in larvae and adults, but cannot rule out the possibility of defects or delays early in development, for which the animal might compensate later. We analyzed NMJ synapse ultrastructure after HPF/FS of embryonic NMJs (Fig. 4). Even in the complete absence of neurexin (*Df(3R)Exel6191*), NMJ synapses appeared relatively normal, except as also noted in larval neurexin mutant NMJs [37], pre and postsynaptic densities were incompletely apposed in the absence of neurexin. The number of synaptic vesicles, dense core vesicles, active zone diameter, and postsynaptic density (PSD) diameter in *nrx* mutants were all statistically indistinguishable from wildtype NMJ synapses. However, the overall synapse density per NMJ appeared lower without neurexin. For example, serial sectioning of NMJs from WT animals revealed synapses in 12 in 38 sections (32%). In contrast, only 9 of 104 NMJ sections (9%) from *Df(3R)Exel6191* mutant embryos had synapses.

**Figure 5:**
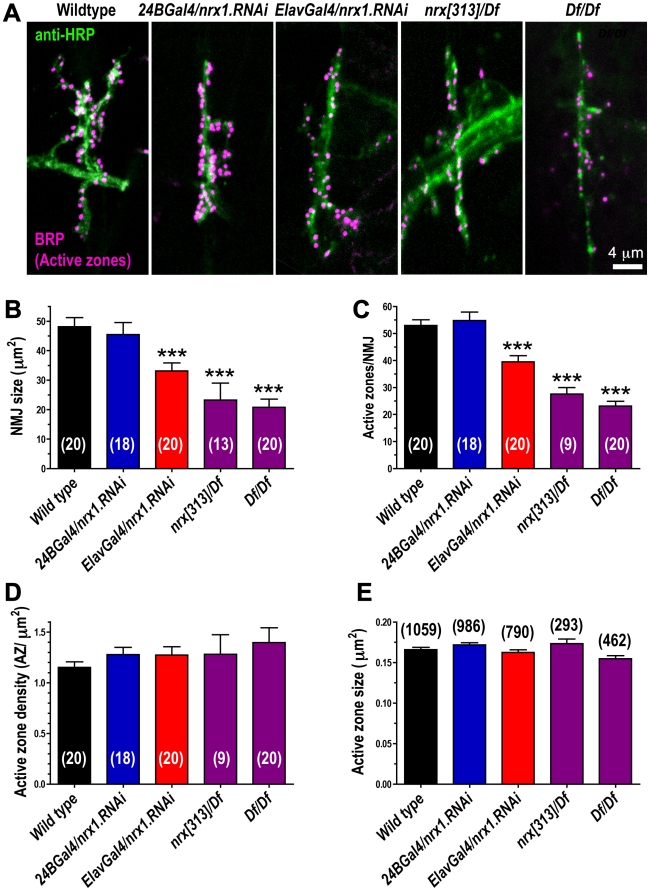
Loss of presynaptic neurexin leads to smaller NMJs with fewer active zones in embryo. A: representative confocal images of embryonic muscle 6/7 NMJs stained with antibodies against bruchpilot (BRP; magenta) to mark active zones, and the presynaptic neuronal membrane marker anti-HRP (green). B: Quantification of NMJ size, measured from anti-HRP immunofluorescence. C: Number of active zones per NMJ, quantified by counting BRP puncta in each NMJ. D: Active zone density, measured by dividing number of active zones in each NMJ by the area of each NMJ. E: Active zone size, quantified by measuring area of every BRP punctum in each 6/7 NMJ. Numbers in parentheses represent either the number of animals (B-D) or the number of active zones (E; measured from 9-20 animals) examined for each measurement.

To explore the apparent loss of synapses per NMJ further, we stained embryonic NMJs using anti-HRP antibodies (which allow visualization of the entire presynaptic NMJ arborization), and anti-bruchpilot (BRP) antibodies, which label individual active zones. As shown in Figs. 5A–C, NMJ size and number of active zones per NMJ were both significantly reduced in the absence of neurexin (*nrx[313]/Df(3R)Exel6191*). RNAi knockdown of neurexin in presynaptic neurons (*ElavGal4/nrx.RNAi*) also reduced NMJ size and active zones per NMJ (Fig. 5A–C). Postsynaptic knockdown of neurexin (*24BGal4/nrx.RNAi*) had no detectable effect on NMJ size or number of active zones per NMJ (Fig. 5A–C). Neither active zone density within the NMJ or active zone size were affected by loss of neurexin (Fig. 5A, D–E). We conclude, based on our EM and immunohistochemical data, that loss of neuronal neurexin reduces active zone formation during embryogenesis.

**Figure 6:**
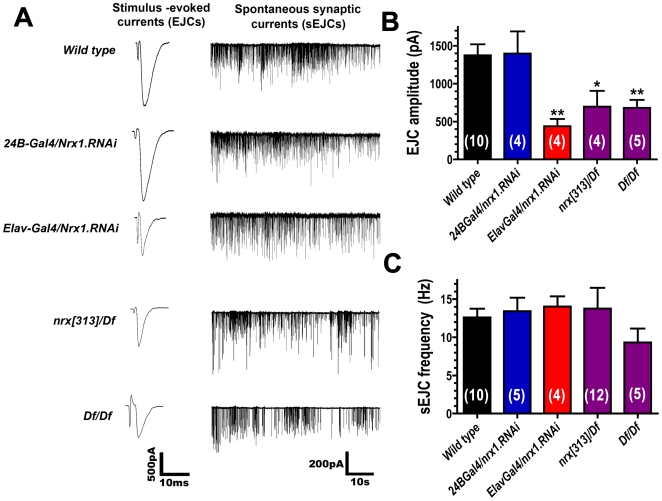
Loss of presynaptic neurexin in embryo disrupts synaptic transmission. A: Representative electrophysiological recordings of stimulus evoked synaptic currents (generated by electrical stimulation of presynaptic motor nerves), and spontaneous excitatory junction currents (sEJCs), from wild type embryonic NMJs and after disruption of neurexin expression. B: Quantification of EJC amplitude. C: Quantification of sEJC frequency. Numbers in parentheses represent the number of animals from which each measurement was made.

**Figure 7:**
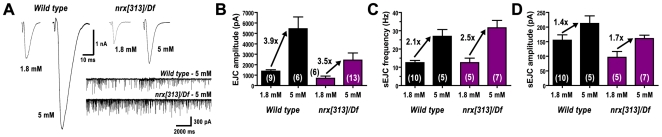
Calcium dependence of embryonic synapse transmission in neurexin mutants. A: EJCs recorded from both wildtype and neurexin mutant animals were significantly larger in 5 mM extracellular calcium, compared to 1.8 mM extracellular calcium. sEJC frequencies were also increased in 5 mM calcium (compare traces to Fig. 6). B: Quantification of EJC amplitudes from wildtype and neurexin mutant animals, in normal (1.8 mM) and high (5 mM) calcium. High calcium caused EJC amplitudes in both genotypes to increase approximately 3.7-fold. C: Quantification of sEJC frequency from wildtype and neurexin mutant animals, in normal (1.8 mM) and high (5 mM) calcium. High calcium caused sEJC frequency in both genotypes to increase approximately 2.3-fold. D: Quantification of sEJC amplitude (which represents, in part, multivesicular events) from wildtype and neurexin mutant animals, in normal (1.8 mM) and high (5 mM) calcium. High calcium caused sEJC amplitude in both genotypes to increase approximately 1.55-fold. The calcium-dependent increases *within* each genotype were statistically significant for all measurements (B-D), but the *fold increase between genotypes* was not statistically significant for any experiment (B-D), suggesting that WT and neurexin mutants functionally respond the same way to increased extracellular calcium. Numbers in parentheses represent the number of animals from which each measurement was made.

We recorded spontaneous and evoked synaptic currents from patch-clamped muscle 6 NMJs in embryos — the same junctions that we analyzed immunohistochemically (Fig. 5). As shown in Fig. 6, electrical stimulation of presynaptic motor nerve axons in wildtype embryos trigger large (∼1500 pA) excitatory junction currents (EJCs). EJC amplitudes were significantly reduced in neurexin mutants (*nrx[313]/Df and Df/Df*; Fig. 6). Presynaptic expression of *neurexin* RNAi (*ElavGal4/nrx.RNAi*) also reduced EJC amplitude (Fig. 6). Postsynaptic expression of *neurexin* RNAi (*24BGal4/nrx.RNAi*) had no effect on EJC amplitude (Fig. 6). The frequency of spontaneous excitatory junction currents was not significantly altered in any genotype (Fig. 6). To check whether reduced EJC amplitudes might be due to reduced calcium sensitivity for neurotransmitter release, we compared EJC amplitudes recorded from wild type and neurexin mutants (*nrx[313]/Df*) in two different calcium concentrations (Fig. 7). Both genotypes showed similar calcium sensitivity (Fig. 7). These results suggest that reduced EJC amplitudes are a consequence of fewer synapses in neurexin mutants.

We also explored whether neurexin might regulate expression, clustering, or function of postsynaptic glutamate receptors. *Drosophila* embryonic/larval NMJs contain two molecularly, biophysically, and pharmacologically distinct subtypes of postsynaptic glutamate receptor: ‘A-type’ and ‘B-type’. The in vivo molecular composition of both receptor subtypes has been determined [40], [41], [42], [43], [44], [45]. A-type receptors contain the subunits GluRIIA, GluRIIC, GluRIID, and GluRIIE. B-type receptors contain the subunits GluRIIB, GluRIIC, GluRIID, and GluRIIE. Although there is some mixing of glutamate receptor subtypes during larval development [43], A- and B-type receptors in embryonic NMJs are spatially segregated into homotypic clusters that are differentially associated with the postsynaptic cytoskeleton [46], [47].

**Figure 8:**
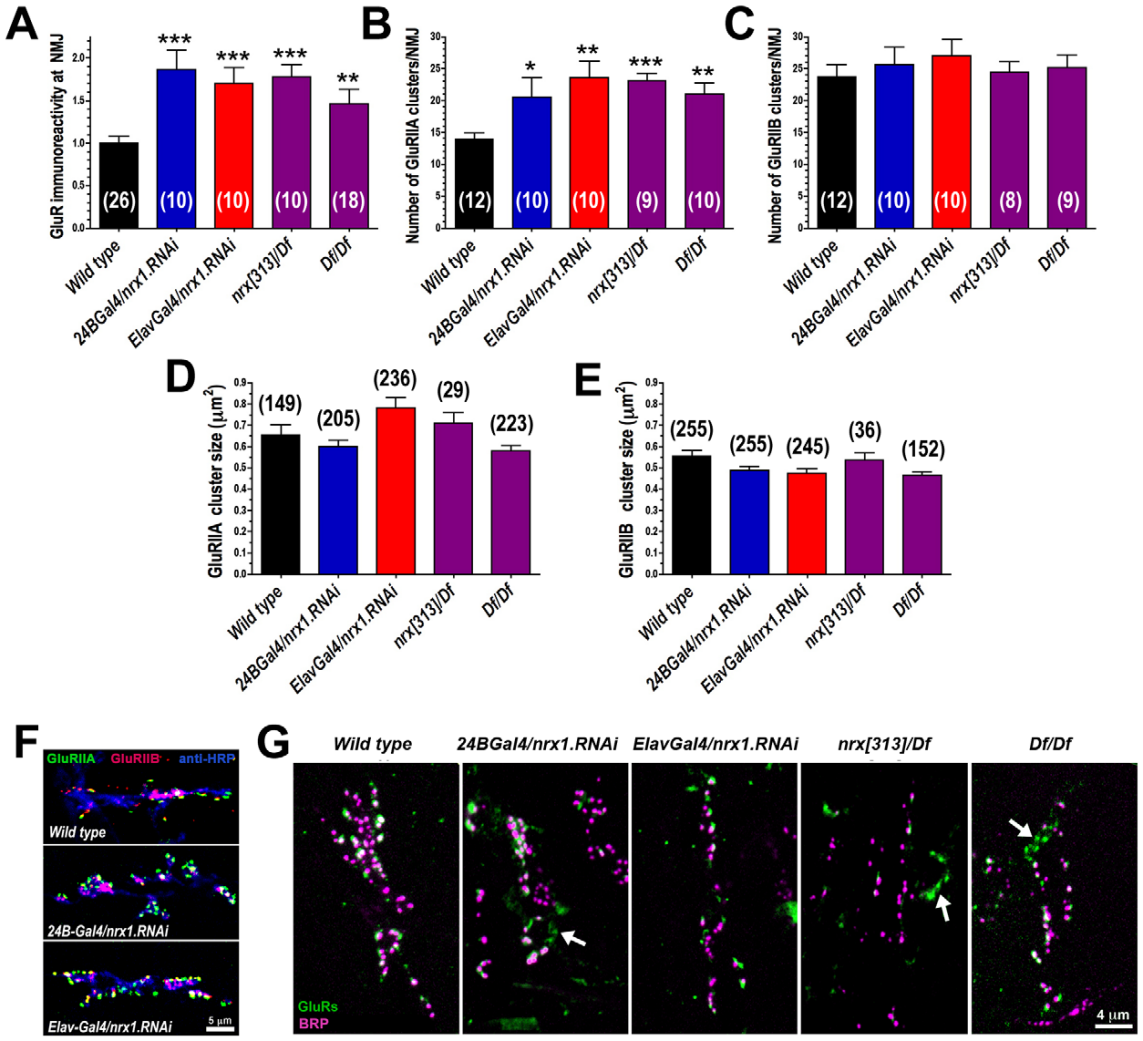
Abundance and localization of postsynaptic glutamate receptor protein. A: Quantification of total glutamate receptor immunoreactivity in embryonic muscle 6/7 NMJs, measured from anti-GluRIIC immunoreactivity. B: Number of A-type glutamate receptor clusters per NMJ, measured by counting number of anti-GluRIIA puncta in each muscle 6/7 NMJ. C: Number of B-type glutamate receptor clusters per NMJ, measured by counting number of anti-GluRIIB puncta in each muscle 6/7 NMJ. D: A-type glutamate receptor cluster area, measured from anti-GluRIIA immunoreactivity. E: B-type glutamate receptor cluster area, measured from anti-GluRIIB immunoreactivity. F: Loss of either postsynaptic (*24B-Gal4/nrx1.RNAi*) or presynaptic (*Elav-Gal4/nrx1.RNAi*) neurexin triggers a shift in the ratio of A-type (green) to B-type (magenta) glutamate receptors, such that A-type glutamate receptors become visibly more abundant (note relative increase in green). G: Loss of either pre or postsynaptic neurexin led to formation of ectopic (nonsynaptic) glutamate receptor clusters, shown here as GluRIIC immunoreactivity (green) that does not overlap with immunoreactivity for the active zone marker bruchpilot (BRP). N (in parentheses for each measurement) represents number of animals (A-C) or number of clusters (D-E).

We measured significantly increased GluRIIC immunoreactivity in embryonic NMJs of neurexin mutants (*nrx[313]Df and Df/Df*), and after presynaptic or postsynaptic RNAi knockdown of neurexin (Fig. 8A). This change could be due to an increase in A-type receptors, B-type receptors, or both A- and B-type receptors. To determine which was the case, we used GluRIIA and GluRIIB-specific antibodies. As shown in Fig. 8B, GluRIIA immunoreactivity was increased in neurexin mutants (*nrx[313]Df and Df/Df*), and after presynaptic or postsynaptic RNAi knockdown of neurexin (Fig. 8B). This increase was not accompanied by a change in the size of individual A-type receptor clusters (Fig. 8D), and there was no change in GluRIIB immunoreactivity (Fig. 8C, E). Because loss of neurexin caused the number of GluRIIA clusters to increase without altering the number of GluRIIB clusters, the A- to B-type receptor ratio in PSDs was shifted in favor of A-type – an effect visible when co-staining with GluRIIA and GluRIIB antibodies (Fig. 8F). We also observed ‘ectopic’ GluRIIA immunoreactivity that did not colocalize with that of the active zone protein bruchpilot (BRP) [48] (Fig. 8G). Postsynaptic GluRIIA immunoreactivity normally colocalizes with presynaptic BRP [49].

**Figure 9:**
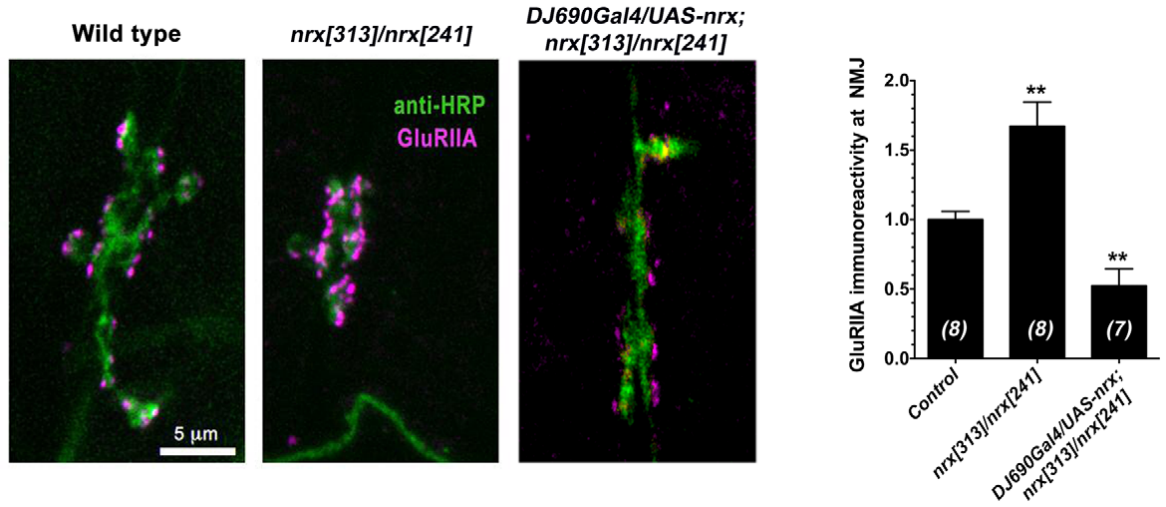
Changes in GluRIIA after rescuing neurexin expression in null mutant. A: Representative confocal micrographs of muscle 6/7 NMJs stained with antibodies against the neuronal membrane marker anti-HRP (green), or GluRIIA (magenta), in wild type embryos, neurexin mutant (*nrx[313]/nrx[241]*) embryos, and in embryos after expression of neurexin cDNA in a neurexin null mutant background (*DJ690Gal4/UAS-nrx; nrx[313]/nrx[241]*). B: Quantification of total GluRIIA immunoreactivity in each genotype.

The results above suggest that neurexin is necessary for proper GluRIIA abundance and localization. To test whether neurexin is sufficient for proper GluRIIA abundance, we cloned a neurexin cDNA and used it to express GFP-tagged neurexin. When this neurexin transgene was overexpressed (both pre and postsynaptically) in a neurexin null mutant background (*DJ690Gal4/UAS-nrx;nrx[313]/nrx[241]*), GluRIIA immunoreactivity in NMJs was significantly reduced, compared to controls (Fig. 9).

**Figure 10:**
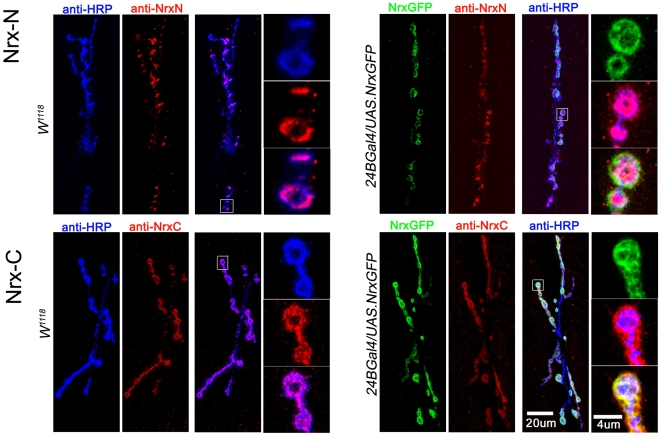
Postsynaptically-expressed neurexin::GFP localizes to NMJs and the C-terminal GFP tag is intracellular. Left panels: N- (top) and C- (bottom) terminal antibody staining in control larval NMJs. Right panels: N- (top) and C- (bottom) terminal antibody staining in larval NMJs after postsynaptic expression of GFP-tagged neurexin. Antibodies raised against a neurexin C-terminal epitope more perfectly overlap fluorescence from GFP-tagged neurexin, compared to immunoreactivity from antibodies raised against a neurexin N-terminal epitope (See methods). This is consistent with the fact that the C-terminal of neurexin is tagged with GFP, and also contains the epitope for the C-terminal antibody. In addition, N-terminal immunoreactivity is more apparent in presynaptic terminals whereas C-terminal immunoreactivity is more apparent in postsynaptic muscle, suggesting tissue-specific differences in epitope accessibility, possibly due to tissue-specific differences in interactions between neurexin and other proteins.

**Figure 11:**
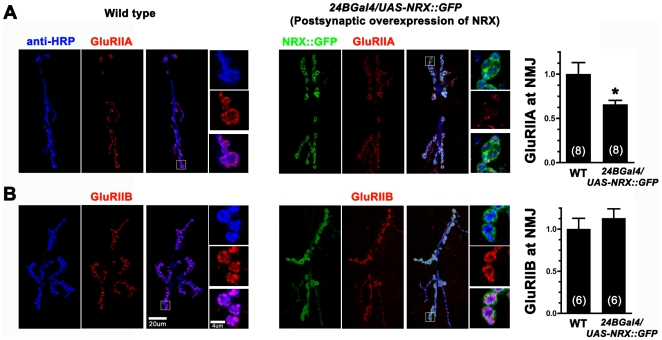
Postsynaptic overexpression of neurexin reduces the number of A-type, but not B-type receptors in larval NMJs. A: Anti-GluRIIA immunoreactivity is increased in *24BGal4/UAS-NRX::GFP* larval NMJs, which overexpress GFP-tagged neurexin specifically in postsynaptic muscle. B: Anti-GluRIIB immunoreactivity is unchanged after postsynaptic overexpression of neurexin.

To test explicitly whether postsynaptic neurexin plays a role in GluRIIA abundance, and to confirm and detail a previous suggestion that neurexin also suppresses GluRIIA in larvae [37], we overexpressed neurexin exclusively in postsynaptic muscle in a wildtype background and quantified GluRIIA immunoreactivity in larval NMJs (Figs. 10,11). GFP-tagged neurexin expressed exclusively in postsynaptic muscle localized to the NMJ, and GFP fluorescence overlapped with anti-neurexin antibody staining (Fig. 10). GluRIIA immunoreactivity in the larval NMJ was reduced after postsynaptic overexpression of neurexin (Fig. 11). In contrast, GluRIIB immunoreactivity remained unchanged (Fig. 11).

**Figure 12:**
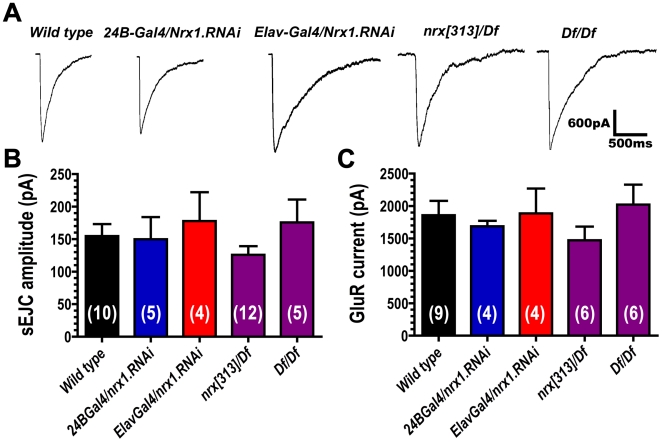
Loss of neurexin causes little change in the number of functional postsynaptic receptors. A: Representative electrophysiological recordings of glutamate-gated currents triggered by pressure ejection of 1 mM glutamate onto patch-clamped embryonic muscle 6. B: Quantification of spontaneous synaptic current (sEJC) amplitude, measured from recordings such as those shown in figure 6A. C: Quantification of glutamate-gated current amplitude (elicited by pressure ejection of 1 mM glutamate onto NMJs). Numbers in parentheses represent the number of animals from which each measurement was made.

To test whether the increased GluRIIA and GluRIIC immunoreactivity observed in neurexin mutants might correlate with increased receptor function, we pressure ejected glutamate onto NMJs and recorded glutamate-gated currents after loss and knockdown of neurexin in embryos (Fig. 12). There was no change in the amplitude of glutamate-gated currents after loss of neurexin (Fig. 12A, C). However, glutamate-gated currents in neurexin mutants tended to be highly variable and often prolonged (Decay time constant 95% confidence intervals: WT = 168–302 ms; *24BGal4/nrx.RNAi* = 92–596 ms; *ElavGal4/nrx.RNAi* = 478–1033 ms; *nrx[313]/Df* = 43–716; *Df/Df* = 535–1297 ms). These data, along with the data from Figures 6 and 8, suggest that pre or postsynaptic loss of neurexin causes an increase in A-type glutamate receptor expression, but these excess receptors may not function properly or be localized directly opposite presynaptic sites of neurotransmitter release.

## Discussion

The first complete knockout of neurexin function was achieved in *Drosophila*, and Zeng et al (2007) provided the first report of *Drosophila* neurexin mutants. Using western blots and immunohistochemistry, Zeng et al (2007) showed that neurexin mutants had reduced brp expression in larval brain, and reduced synapse density in adult brain [36]. Both results are consistent with the idea that neurexin promotes synapse formation or maintenance, as previously argued by many studies in mammalian cells and alpha neurexin mouse mutants [30], [31], [35], [50], [51], [52], [53], [54]. Li et al (2007) subsequently provided a detailed examination of neurexin mutant larval NMJs, and showed that larval NMJ arborizations were smaller, similar to what we describe here in embryos (Fig. 5). Sun et al (2009) also reported smaller NMJs in larval neurexin mutants. However, Li et al (2007) reported a large increase in the number of presynaptic densities (‘T-bars’) and boutons (with no obvious decrease in active zone density per bouton) in neurexin mutant larval NMJs, which contrasts with the idea that neurexin promotes synaptogenesis and our observation that the number of presynaptic densities (measured either by EM or brp staining) is reduced in neurexin mutant embryonic NMJs. The proliferation of active zones observed in neurexin mutant larvae may therefore represent developmental compensation for reduced muscle excitation. Increased active zone proliferation during larval development has previously been observed in other mutants with reduced NMJ transmission [55].

Li et al (2007) also described apparent disruptions in cell adhesion between presynaptic neurons and postsynaptic muscle in larval neurexin mutant NMJs, which appeared as widened synaptic clefts visible by electron microscopy [37]. We observed no such changes in any of the dozens of sections from 14 separate embryonic NMJs that we examined. The comparison suggests that these ultrastructural changes may occur during larval development rather than initial synapse formation.

Mouse alpha neurexin mutants show dramatic defects in calcium-dependent neurotransmitter release [50], [53]. *Drosophila* neurexin mutants also show reductions in neurotransmitter release, both at the larval stage [37], [39], and in embryos (Fig. 6). In embryos, decreased neurotransmission in neurexin mutant NMJs appears entirely attributable to the decrease in NMJ active zone number that we observed (Fig. 5). In older stages, and in mammals, some of the decreased neurotransmitter release is attributed to defects in calcium-secretion coupling [37], [50], [53].

Miniature postsynaptic potentials are larger in larval neurexin mutant NMJs [37], [39]. In contrast, we observed no change in embryonic neurexin mutant sEJC amplitude, and no change in the size of individual receptor cluster sizes that typically go along with such changes. Instead, we saw an increase in the number of postsynaptic receptor clusters (Fig. 8) without any corresponding increase in presynaptic active zones. Our data, taken together with larval results, suggests that loss of neurexin initially causes an increase in nonfunctional postsynaptic receptor clusters. During larval development, many of these receptor clusters then become paired with presynaptic active zones, so that there is an increase in synapse number as described by Li et al (2007). At this stage, increased receptor expression is manifest as an increase in individual receptor cluster sizes and miniature postsynaptic potentials. Besides the insights into neurexin function, this is interesting because it suggests that glutamate receptors in embryos can semi-autonomously form clusters, but receptors in larvae are preferentially added to pre-existing synapses.

Importantly, our data demonstrate that neurexin mutant glutamate receptor phenotypes, at least in embryos, are restricted to A-type receptors. This suggests a mechanism. *Drosophila* A-type, but not B-type, glutamate receptors depend on postsynaptic F-actin for localization/stabilization [47], and this process involves a direct interaction between the C-terminus of GluRIIA and the *Drosophila* 4.1 protein ‘coracle’ [47]. The intracellular C-terminus of mammalian neurexin has been shown to bind to the PDZ domain protein CASK [26], and interactions between mammalian neurexin and CASK in combination with 4.1 protein have been shown to nucleate F-actin assembly [56]. CASK knockout mice are lethal but show no dramatic synaptic alterations except increased neuroligin protein levels and higher spontaneous event frequency at glutamatergic synapses [57], consistent with our data and a subtle role in synaptic protein organization. The *Drosophila* genome encodes one CASK homolog, which interacts with neurexin to regulate walking behavior and neuromuscular transmission [39], [58], [59]. *Drosophila* neurexin may therefore work with CASK and coracle (4.1) to regulate A-type glutamate receptor organization via actin rearrangements. Mammalian beta neurexin also appears to regulate AMPA receptor abundance in a subunit-specific manner [60], and alpha neurexin appears to regulate NMDA but not AMPA receptor function [51]. But in these cases receptors are recruited by the presence of neurexin rather than repressed, as is seen in *Drosophila*. This added complexity may be due to differing molecular functions of neurexin within pre and postsynaptic cells. Interestingly, complete loss of neurexin in both pre and postsynaptic cells led to the same 150–200% increase in GluRIIA as loss of either pre or postsynaptic neurexin (Fig. 8A). The increase in GluRIIA immunoreactivity observed after loss of neurexin on any side of the synapse might therefore represent a physiological limit, or (more likely) *either* pre or postsynaptic neurexin is sufficient to suppress GluRIIA in WT animals.

The most controversial suggestion provided by our data is the possibility that neurexin in *Drosophila* NMJs might be present in postsynaptic muscle, where it appears to contribute (along with presynaptic neurexin) to formation of proper glutamate receptor clusters in embryos. Previous studies did not report neurexin expression in muscles [36], [37]. However, the embryonic cuticle forms at approximately the same time in development as the body wall muscles, and suppresses the ability of RNA probes to enter tissues, making it difficult to detect expression of muscle genes that are expressed late in embryonic development. Indeed, we also initially found it difficult to unambiguously detect neurexin expression in body wall muscles of intact embryos by in situ hybridization. Previous studies did not test explicitly whether postsynaptic neurexin affected postsynaptic receptor clustering, but agree with our result that postsynaptic neurexin does not affect presynaptic differentiation or function. Taniguchi et al (2007) also suggested that neurexin might function postsynaptically in mammalian cells. Specifically, they suggested that postsynaptic neurexin could interact with postsynaptic neuroligin to reduce transsynaptic neurexin-neuroligin interactions [61]. This hypothesis does not quite fit with our data, however, since we observed that knockdown of presynaptic neurexin and postsynaptic neurexin had the same receptor phenotype. If postsynaptic neurexin counteracted presynaptic neurexin function, one would expect postsynaptic neurexin knockdown to have the opposite effect of presynaptic neurexin knockdown. The simplest hypothesis that allows for all the results is that presynaptic neurexin works via transynaptic interactions (with neuroligin or other proteins), while postsynaptic neurexin works primarily via intracellular C-terminal interactions to regulate receptor clustering, as hypothesized above. Note that these postsynaptic neurexin intracellular interactions do not exclude any previously demonstrated interactions between the intracellular C-terminus of postsynaptic neuroligin and other proteins, including PSD-95 [32], [33], [34], [35], [62].

## Materials and Methods

### In situ hybridization

To produce the in situ probes pSL *nrx-1* was digested with *EcoR*I and *Xba*I and the fragment ligated into pBluescript II KS+. The final vector (pKs+ Nrx-1) was cut with *EcoR*I to make the antisense probe with T7 (5′-AAT ACG ACT CAC TAT AG-3′). The sense control probe was made with T3 (5′- ATT AAC CCT CAC TAA AGG GA-3′) after cutting pKS+ Nrx-1 with *Xba*I. In situs were performed following the Berkeley *Drosophila* Genome Project (BDGP) standard methods (http://www.fruitfly.org/about/methods/index.html). Following hybridization, embryos were manually staged and dissected to examine neurexin expression. Dissections were performed as for confocal imaging and electrophysiology (described below).

### Immunohistochemistry and confocal microscopy

For immunohistochemistry and confocal microscopy, animals were manually dissected and fixed in Bouin's fixative as previously described [46], [47]. Neurexin ‘N-terminus’ polyclonal antibodies were raised in rabbits against the synthetic neurexin-specific peptide ELRLLPAQRTSTSAFESPDLR and affinity purified by Open Biosystems (Huntsville AL). Neurexin ‘C-terminus’ polyclonal antibodies were generated and affinity purified the same way, but the neurexin - specific peptide used as an antigen was QPKAKKRDSKDVKEWYV. Anti-NRX antibodies were used at 1∶500 in late stage embryos (22–24 h after egg laying (AEL) at 25C, temporally and morphologically staged). Mouse monoclonal anti-GluRIIA, from a cell line made by Christoph Schuster and available from University of Iowa's Developmental Studies Hybridoma Bank (DSHB), was used at 1∶100 [63]. Rabbit polyclonal anti-GluRIIB and rabbit polyclonal anti-GluRIIC, first described in Marrus et al (2004) and subsequently replicated in the lab of D.E.F., were used at 1∶2000 [43]. Mouse NC82 (anti-bruchpilot), a gift from Eric Buchner, was used at 1∶100 [48]. Fluorescently-conjugated anti-HRP (Jackson Immuno laboratories, West Grove PA) was used at 1∶200. Immunoreactivity was visualized using FITC-, TRITC-, or CY5-conjugated goat anti-mouse/rabbit secondary antibodies (Jackson Immuno Laboratories), used at 1∶400–1∶2000. All images of NMJs are from ventral longitudinal muscles 6 and 7 in abdominal segments 3–4. Control and experimental preparations were always stained and imaged in parallel.

Images were captured using an Olympus Fluoview FV500 laser scanning confocal system. Quantifications of staining intensity were performed as previously described (46). Briefly, fluorescence intensity of relevant structures from unaltered unsaturated confocal maximum intensity projections was measured by manually selecting the region of interest in imageJ and measuring mean pixel intensity of that region. To control for differences in individual preparation immunoreactivity, excitation, fluorescence attenuation, and detection, we then subtracted mean pixel intensity of a similarly-sized region of unstained ‘background’ in the same fluorescence wavelength channel in the same image. Immunohistochemical measurement of active zone and GluR cluster size was also done as previously described [63], [46], [47]. Briefly, ImageJ software was used to manually outline every puncta visible on a 6/7 NMJ. The puncta area (in pixels) was then measured and converted to square micrometers based on pixel size values computed for each image by Fluoview confocal software, which automatically records image resolution, microscope objective, and ‘zoom’ factor for each image. Muscle 6/7 NMJ sizes were similarly measured, using synaptic anti-HRP fluorescence. Photoshop CS2 was then used to crop images and adjust contrast for final display.

### Electron microscopy

For electron microscopy, genotyped *Drosophila* eggs were dechorionated with bleach and manually devitellinated before immersion in a bacteria slurry for HPF/FS. High pressure freezing, freeze substitution (HPF/FS), and transmission electron microscopy (TEM) were performed as previously described for *C. elegans* [64], [65]. Briefly: Fixed whole embryos were thin sectioned (40–50 nm) onto formvar-coated copper grids and counterstained using 2.5% uranyl acetate and Reynold's lead citrate. Images were collected from late embryonic (22–24 h AEL) body wall NMJs in abdominal segments, selected based on proximity to cuticle midway along the longitudinal axis of the embryo. For immuno EM analysis, animals were fixed with 0.1% potassium permanganate for 72 hours and embedded in Lowicryl for 60 hours under UV light. Thin (∼50 nm) sections from Lowicryl embedded samples were collected as ribbons on formvar-coated nickel slot grids and immuno-gold labeled in a similar fashion to that described previously [66]. The neurexin antibody was used at a dilution of 1∶30. Anti-rabbit-10 nm gold bead conjugated antibodies were diluted 1∶150.

### Generation of *UAS-nrx1.RNAi*

*UAS-nrx.RNAi* flies were generous gifts from Mary Gilbert and Vanessa Auld (University of British Columbia, Canada). The *UAS-nrx.RNAi* transgene was created as follows: A 2.1 kb genomic fragment of *neurexin 1* was amplified using PCR from *EP(3)0709* genomic DNA using oligonucleotides RINrx (RINrx: 5′-GAATTCTAACCGTGGATGACTCCTTC-3′) and XhoNrx (XhoNrx: 5′-CTCGAGCGAGAAGATCAGATCGTCCA-3′), then subcloned into pGEM-T using standard methods. A genomic fragment was subsequently excised from pGEM-T with SalI (5′ end) and XhoI (3′ end) and subcloned directionally into pBS KS+ (Stratagene). This genomic fragment was then excised from pBS with XbaI (5′end) and XhoI (3′ end) and directionally subcloned into an intermediate RNAi plasmid provided by Genetic Services Inc (Sudbury, MA, USA), to generate the tail-to-tail pNrxRNAi plasmid which was then transformed *w1118 Drosophila* embryos. Ten viable transformed lines were produced. All experiments described in this manuscript used line #5.

### Neurexin mutant alleles

Many of the experiments in this study used previously-undescribed *nrx[313]* mutants. This mutation (and several other alleles) was generated by mobilization of *P{Mi}Nrx-1{MB00002}*, using standard methods (50, 51), with the exception that we used the *PhsILMiT* transposase source required for minos mobilization. *PhsILMiT* flies were generously provided by the lab of Dr. H. Bellen (Baylor College of Medicine), whose lab also produced and provided *P{Mi}Nrx-1{MB00002}* flies as part of the ongoing BDGP Gene Disruption Project (23). The Minos element and PhsILMiT transgenes were provided by Minos Biosystems Ltd (Glasgow, Scotland). Neurexin mutants, including *nrx[313]*, were selected after *P{Mi}Nrx-1{MB00002}* mobilization based on loss/alteration in minos element eGFP expression (indicating that the transposon had changed location) and loss of neurexin immunoreactivity.*Df(3R)Exel6191* mutants were obtained from the Bloomington *Drosophila* Stock Center. *nrx[241]* mutants, described in Li et al (2007), were a generous gift from Manzoor Bhat.

We sequenced the *neurexin* genomic region in wildtype embryos, homozygous *nrx[313]* mutants, and flies in which *P{Mi}Nrx-1{MB00002}* was excised with no phenotype (e.g. precise excision animals). Sequences from these three genotypes were compared to each other and to the reference sequence from the *Drosophila* genome project. As expected, *P{Mi}Nrx-1{MB00002}* was completely excised in *nrx[313]* mutants. Surprisingly, we detected only a relatively small genomic alteration in *nrx[313]* mutant chromosomes: a 10 bp deletion in *neurexin* intron #7, approximately 3 kb from the original *P{Mi}Nrx- 1{MB00002}* insertion site. Genscan (52) predicted that this deletion (loss of CAGCTGCAAC) would cause no change in *neurexin* splicing. However, inspection with VISTA comparative genomics tools (53) revealed that the *nrx[313]* deletion removes a stretch of very highly conserved intronic sequence. This sequence stretch has 95–100% base pair conservation between different *Drosophila* species -- higher than that found in most *neurexin* exons. Thus, it likely represents a previously unidentified essential regulatory sequence. To confirm that this small deletion represents a true loss-of-function mutation for *neurexin*, we performed complementation tests with two other molecularly characterized *neurexin* null alleles, *Df(3R)Exel6191* and *nrx[241]*, and used neurexin antibodies to quantify neurexin protein immunoreactivity in the NMJ of each genotype (Fig. 3). *Df(3R)Exel6191* removes the entire neurexin gene region; *nrx[241]* is a precise deletion of the neurexin coding region (21). As expected, *nrx[313]* failed to complement either of these other alleles, either with regard to NMJ neurexin staining or any of the major neurexin mutant phenotypes. *nrx[313]* is therefore unambiguously a neurexin null allele, based on both genetic and phenotypic criteria.

### Electrophysiology

Patch clamp electrophysiology on late embryonic (22–24 h AEL) NMJs was performed as previously described [47], [67]. Briefly, muscle 6 was whole-cell voltage clamped at −60 mV in standard *Drosophila* saline (135 mM NaCl, 5 mM KCl, 4 mM MgCl_2_, 1.8 mM CaCl_2_, 72 sucrose, and 5 TES, pH 7.2). Where noted, extracellular calcium was increased to 5 mM. The patch pipette solution contained (in mM): 120 KCl, 20 KOH, 4 MgCl_2_, 0.25 CaCl_2_, 5 EGTA, 36 sucrose, and 5 TES. Evoked synaptic currents were triggered by 5–10 V electrical stimulation of the appropriate segmental nerve using a suction electrode, as previously described (46). Glutamate-gated currents were evoked by pressure ejection of 1 mM glutamate onto NMJs using small glass pipettes and a picospritzer II, as previously described [68].

### Statistics

Statistical significance was determined using unpaired Student t-tests (for normally distributed data) or nonparametric Mann Whitney tests (when post-hoc F-tests determined that variances were significantly different). In all figures, asterisks indicate statistical significance: *** = p<0.001, ** = p<0.01, * = p<0.05. N for each measurement is indicated in each figure (N = number in parentheses). In all cases, unless stated otherwise, N = the number of different animals from which measurements were taken. Error bars represent standard error of the mean (S.E.M.).
